# Oscillometric measurement of systolic and diastolic blood pressures validated in a physiologic mathematical model

**DOI:** 10.1186/1475-925X-11-56

**Published:** 2012-08-22

**Authors:** Charles F Babbs

**Affiliations:** 1 Department of Basic Medical Sciences, Weldon School of Biomedical Engineering, Purdue University, 1426 Lynn Hall, West Lafayette, IN, 47907-1246, USA

## Abstract

**Background:**

The oscillometric method of measuring blood pressure with an automated cuff yields valid estimates of mean pressure but questionable estimates of systolic and diastolic pressures. Existing algorithms are sensitive to differences in pulse pressure and artery stiffness. Some are closely guarded trade secrets. Accurate extraction of systolic and diastolic pressures from the envelope of cuff pressure oscillations remains an open problem in biomedical engineering.

**Methods:**

A new analysis of relevant anatomy, physiology and physics reveals the mechanisms underlying the production of cuff pressure oscillations as well as a way to extract systolic and diastolic pressures from the envelope of oscillations in any individual subject. Stiffness characteristics of the compressed artery segment can be extracted from the envelope shape to create an individualized mathematical model. The model is tested with a matrix of possible systolic and diastolic pressure values, and the minimum least squares difference between observed and predicted envelope functions indicates the best fit choices of systolic and diastolic pressure within the test matrix.

**Results:**

The model reproduces realistic cuff pressure oscillations. The regression procedure extracts systolic and diastolic pressures accurately in the face of varying pulse pressure and arterial stiffness. The root mean squared error in extracted systolic and diastolic pressures over a range of challenging test scenarios is 0.3 mmHg.

**Conclusions:**

A new algorithm based on physics and physiology allows accurate extraction of systolic and diastolic pressures from cuff pressure oscillations in a way that can be validated, criticized, and updated in the public domain.

## Background

The ejection of blood from the left ventricle of the heart into the aorta produces pulsatile blood pressure in arteries. Systolic blood pressure is the maximum pulsatile pressure and diastolic pressure is the minimum pulsatile pressure in the arteries, the minimum occurring just before the next ventricular contraction. Normal systolic/diastolic values are near 120/80 mmHg. Normal mean arterial pressure is about 95 mmHg [1].

Blood pressure is measured noninvasively by occluding a major artery (typically the brachial artery in the arm) with an external pneumatic cuff. When the pressure in the cuff is higher than the blood pressure inside the artery, the artery collapses. As the pressure in the external cuff is slowly decreased by venting through a bleed valve, cuff pressure drops below systolic blood pressure, and blood will begin to spurt through the artery. These spurts cause the artery in the cuffed region to expand with each pulse and also cause the famous characteristic sounds called Korotkoff sounds. The pressure in the cuff when blood first passes through the cuffed region of the artery is an estimate of systolic pressure. The pressure in the cuff when blood first starts to flow continuously is an estimate of diastolic pressure. There are several ways to detect pulsatile blood flow as the cuff is deflated: palpation, auscultation over the artery with a stethoscope to hear the Korotkoff sounds, and recording cuff pressure oscillations. These correspond to the three main techniques for measuring blood pressure using a cuff [2].

In the palpatory method the appearance of a distal pulse indicates that cuff pressure has just fallen below systolic arterial pressure. In the auscultatory method the appearance of the Korotkoff sounds similarly denotes systolic pressure, and disappearance or muffling of the sounds denotes diastolic pressure. In the oscillometric method the cuff pressure is high pass filtered to extract the small oscillations at the cardiac frequency and the envelope of these oscillations is computed, for example as the area obtained by integrating each pulse [3]. These oscillations in cuff pressure increase in amplitude as cuff pressure falls between systolic and mean arterial pressure. The oscillations then decrease in amplitude as cuff pressure falls below mean arterial pressure. The corresponding oscillation envelope function is interpreted by computer aided analysis to extract estimates of blood pressure.

The point of maximal oscillations corresponds closely to mean arterial pressure [4-6]. Points on the envelope corresponding to systolic and diastolic pressure, however, are less well established. Frequently a version of the maximum amplitude algorithm [7] is used to estimate systolic and diastolic pressure values. The point of maximal oscillations is used to divide the envelope into rising and falling phases. Then characteristic ratios or fractions of the peak amplitude are used to find points corresponding to systolic pressure on the rising phase of the envelope and to diastolic pressure on the falling phase of the envelope.

The characteristic ratios (also known as oscillation ratios or systolic and diastolic detection ratios [8]) have been obtained experimentally by measuring cuff oscillation amplitudes at independently determined systolic or diastolic points, divided by the maximum cuff oscillation amplitude. The systolic point is found at about 50% of the peak height on the rising phase of the envelope. The diastolic point is found at about 70 percent of the peak height on the falling phase of the envelope [7]. These empirical ratios are sensitive however to changes in physiological conditions, including most importantly the pulse pressure (systolic minus diastolic blood pressure) and the degree of arterial stiffness [9,10]. Moreover, a rational physical explanation for any particular ratio has been lacking. Since cuff pressure oscillations continue when cuff pressure falls beneath diastolic blood pressure, the endpoint for diastolic pressure is indistinct. Most practical algorithms used in commercially available devices are closely guarded trade secrets that are not subject to independent critique and validation. Hence the best way to determine systolic and diastolic arterial pressures from cuff pressure oscillations remains an open scientific problem.

The present study addresses this problem with a new approach based upon the underlying physics, anatomy, and physiology. This task requires modeling the cuff and arm and the dynamics of a partially occluded artery within the arm during cuff deflation. A second phase of the problem is the development of a regression procedure for analysis of recorded cuff pressure oscillations to extract model parameters and predict the unique systolic and diastolic pressure levels that would produce the observed cuff pressure oscillations.

### Methods Part 1: Modeling cuff pressure oscillations

#### Model of the cuff and arm

**Figure 1:**
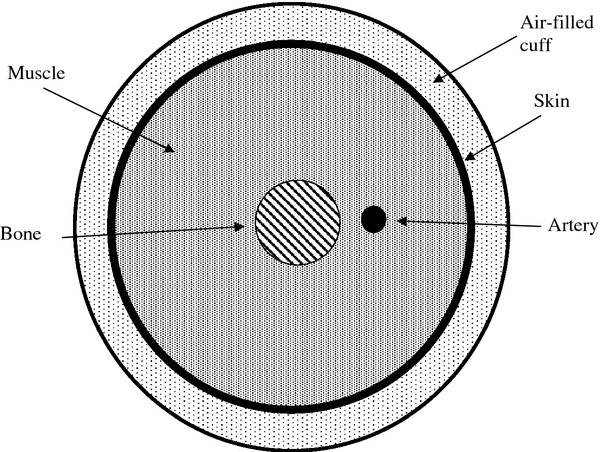
Arrangement of cuff, skin, muscle, bone, and artery for a simple model of the arm during oscillometric blood pressure recording.

As shown in Figure 1, one can regard the cuff as an air filled balloon of dimensions on the order of 30 cm x 10 cm x 1 cm, which is wrapped in a non-expanding fabric around the arm. After inflation the outer wall of the cuff becomes rigid and the compliance of the cuff is entirely due to the air it contains. During an oscillometric run the cuff is inflated to a pressure well above systolic, say 150 to 200 mmHg, and then vented gradually at a bleed rate of r = 3 mmHg / second [11]. Small oscillations in cuff pressure happen when the artery fills and empties with blood as cuff pressure passes between systolic and diastolic pressure in the artery.

Let P_0_ be the maximal inflation pressure of the cuff at the beginning of a run. The pressure is bled down slowly at rate, r mmHg/sec (about 3 mmHg/sec [11]). During the brief period of one heartbeat the amount of air inside the cuff is roughly constant. In addition to smooth cuff deflation, small cuff pressure oscillations are caused by pulsatile expansion of the artery and the corresponding compression of the air in the cuff. One can model the cuff as a pressure vessel having nearly fixed volume, V_0_ − ΔV_a_, where V_0_ is cuff volume between heartbeats and ΔV_a_ is the small incremental volume of blood in the artery beneath the cuff as it expands with the arterial pulse.

To compute cuff pressure oscillations from the volume changes, ΔV_a_, in the occluded artery segment it is necessary to know the compliance of the cuff, C = ΔV/ΔP, which is obtainable from Boyle’s law as follows. Boyle’s law is PV = nRT, where P is the absolute pressure (760 mmHg plus cuff pressure with respect to atmospheric), V is the volume of air within the cuff, n is the number of moles of gas, R is the universal gas constant, and T is the absolute temperature. During the time of one heartbeat, n, R, and T are constants and n is roughly constant owing to the slow rate of cuff deflation. Hence to relate the change in cuff pressure, ΔP to the small change in cuff volume, ΔV, from artery expansion we may write $PV\approx \left(P+\Delta P\right)\cdot \left(V+\Delta V\right)\approx PV+P\Delta V+V\Delta P$, for absolute cuff pressure P. So

(1) $$0\approx P\Delta V+V\Delta P\;\text{and}\;C_{\mathrm{cuff}}=\frac{-\Delta V}{\Delta P}=\frac{\Delta V_{a}}{\Delta P}\approx \frac{V_{0}}{P}\text{.}$$

The negative change in cuff volume represents indentation by the expanding arm when the artery inside fills with blood. The effective cuff compliance, C_cuff_ , or more precisely the time-varying and pressure-varying dynamic compliance of the sealed air inside the cuff, is

(2) $$C_{\mathrm{cuff}}=\frac{dV_{\mathrm{cuff}}}{dP}=\frac{V_{0}}{P+760mmHg}\text{,}$$

with cuff pressure, P, expressed in normal clinical units of mmHg relative to atmospheric pressure. In turn, the time rate of change in cuff pressure is

(3) $$\frac{dP}{dt}=-r+\frac{1}{C_{\mathrm{cuff}}}\frac{dV_{a}\left(t\right)}{dt}≅-r+\left(\frac{P_{0}+760-rt}{V_{0}}\right)\frac{dV_{a}\left(t\right)}{dt}\text{.}$$

In this problem as cuff pressure is slowly released, even as cuff volume remains nearly constant, the dynamic compliance of the cuff increases significantly and its stiffness decreases. Hence a suitably exact statement of the physics requires a differential equation (1a), rather than the constant compliance approximation $P=P_{0}-rt+V_{a}\left(t\right)/C_{\mathrm{cuff}}$. However, equation (1a) may be integrated numerically to obtain a sufficiently exact representation of cuff pressure changes with superimposed cardiogenic oscillations.

#### Model of the artery segment

Next to characterize the time rate of volume expansion of the artery, dV_a_/dt, one can regard the artery as an elastic tube with a dynamic compliance, C_a_, which varies with volume and with internal minus external pressure. The dynamic compliance C_a_ = dV_a_/d(P_a_ – P_o_), where P_t_ = P_a_ – P_o_ is the transmural pressure or the difference between pressure inside the artery and outside the artery. Then

(4) $$\frac{dV_{a}}{dt}=\frac{dV_{a}}{d\left(P_{a}-P_{o}\right)}\cdot \frac{d\left(P_{a}-P_{o}\right)}{dt}≅C_{a}\left(\frac{dP_{a}}{dt}+r\right)\text{,}$$

where the artery “feels” the prevailing difference between internal blood pressure and external cuff pressure, neglecting the small cuff pressure oscillations. The time derivative of arterial pressure can be determined from a characteristic blood pressure waveform and the known rate, r, of cuff deflation. Hence, the crucial variable to be specified next is the dynamic arterial compliance, C_a_.

**Figure 2:**
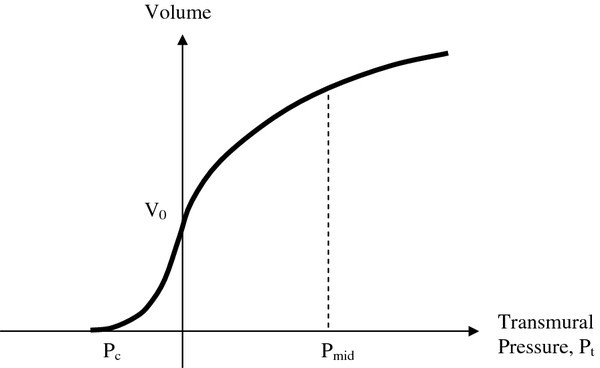
**Hypothetical pressure-volume relationship for an artery including negative transmural pressures and collapse.** P_c_ is collapse pressure, and P_mid_ is normal mid-level arterial pressur.

Specifying the compliance of the artery is more difficult than specifying the cuff compliance, because the pressure across the artery wall during an oscillometric measurement varies over a wide range from negative to positive. Most research studies, such as the classical ones of Geddes and Posey [12], explore only positive distending pressures. A few sources however [9,13] describe pressure-volume functions like the one sketched in Figure 2 for arteries subjected to both positive and negative distending pressure.

For classical biomaterials one can use two exponential functions to model the nonlinear volume vs. pressure relationship over a wide range of distending pressures. Here we shall use two exponential functions: one for negative pressure range and another for the positive pressure range in a manner similar to that described by Jeon et al. [13]. The first exponential function for negative transmural pressure, P_t_<0, is easy to imagine. For the negative transmural pressure domain artery volume $V_{a}=V_{a0}e^{aP_{t}}$ for positive constant, a, and zero pressure volume, V_a0_, of the artery. Here the dynamic compliance is clearly

(5) $$\frac{dV_{a}}{dP_{t}}=aV_{a0}e^{aP_{t}}\;{\text{for P}}_{\text{t}}<0.$$

For the positive transmural pressure domain one can use a similar, but decelerating exponential function [12]. However, there should be no discontinuity at the zero-transmural pressure point (0, V_0_). This means that for positive constant, b, and typically b < a,

(6) $$\frac{dV_{a}}{dP_{t}}=aV_{a0}e^{-bP_{t}}\;{\text{for P}}_{\text{t}}\geq 0.$$

These two exponential functions can be used to characterize the dynamic compliance of the artery model in terms of easily obtained data, including the collapse pressure, P_c_ < 0, defined as the pressure when the artery volume is reduced to 0.1V_a0_ (for example, P_c_ = −20 mmHg) and the normal pressure arterial compliance, C_n_, measured at normal mid-level arterial pressure, P_mid_ , halfway between systolic and diastolic pressure.

Solving for constant, a, we have

(7) $$a=\frac{\ln\left(0.1\right)}{P_{c}}\text{.}$$

Solving for constant, b, we have

(8) $$b=\frac{-\ln\left(\frac{C_{n}}{aV_{a0}}\right)}{P_{\mathrm{mid}}}\text{.}$$

The zero pressure volume, V_a0_, can be known from anatomy if necessary, but as shown later is not needed if one is interested only in the relative amplitude of cuff pressure oscillations.

One can integrate the expressions (2a) and (2b) to obtain analytical volume versus pressure functions similar to Figure 2. Thus for P_t_ < 0

(9) $$V_{a}=V_{a0}+aV_{a0}\int_{0}^{P_{t}}e^{aP_{t}}dP_{t}=V_{a0}e^{aP_{t}}\text{,}$$

and for P_t_ ≥ 0

(10) $$\begin{array}{c} V_{a}=V_{a0}+aV_{a0}\int_{0}^{P_{t}}e^{-bP_{t}}dP_{t}\\ =V_{a0}-\frac{a}{b}V_{a0}\left(e^{-bP_{t}}-1\right)\\ =V_{a0}\left(1+\frac{a}{b}\left(1-e^{-bP_{t}}\right)\right)\text{.} \end{array}$$

**Figure 3:**
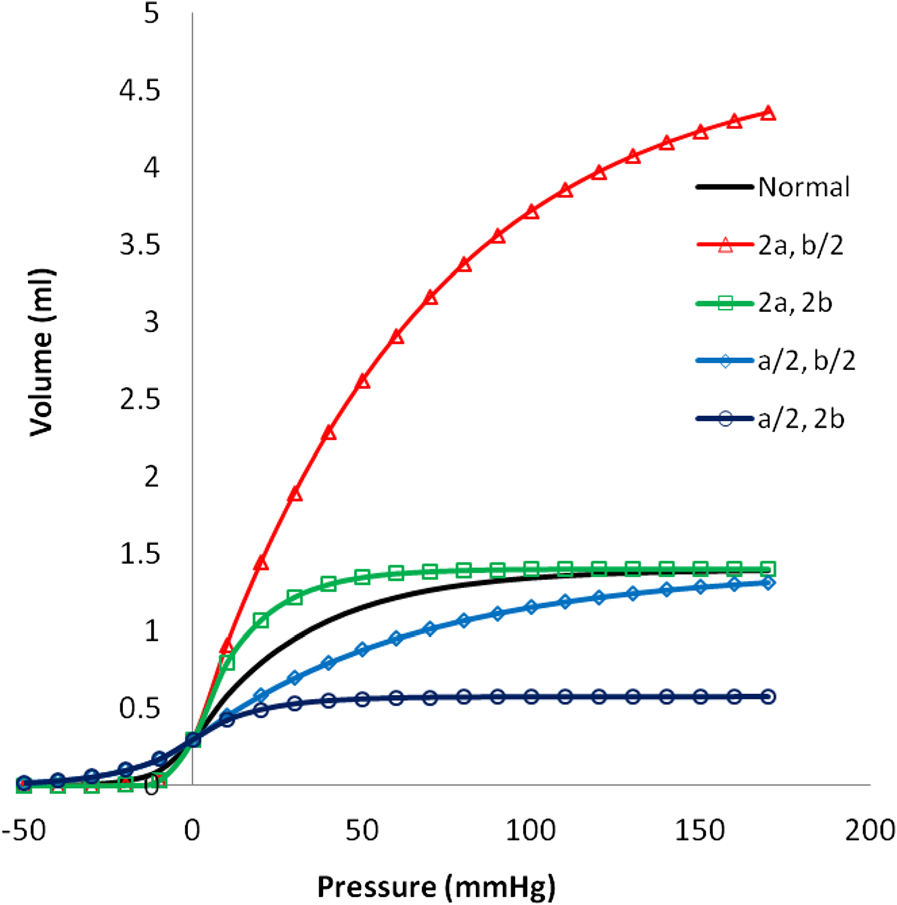
**Representative volume vs. pressure curves for an artery segment over a wide range of positive and negative transmural pressure.** Standard normal variables a = 0.11 mmHg^-1^ , b = 0.03/mmHg^-1^, V_a0_ = 0.3 ml. Variations in shape occur with combinations of increased (2x normal) and decreased (1/2 normal) values of parameters a and b.

Figure 3 shows a plot of the resulting pressure-volume curve for a normal 10-cm long artery segment and constants a and b as described for initial conditions below. The form of the function is quite reasonable and consistent with prior work [9,13]. When bi-exponential constants a and b are varied, a wide variety of shapes for the pressure-volume curve can be represented. When volume changes more rapidly with pressure, the artery is more compliant. When volume changes less rapidly with pressure, the artery is stiffer. Increasing a and b in proportion allows greater volume change for a given pressure change and represents a more compliant artery. Decreasing a and b in proportion reduces the volume change for a given pressure change and represents a stiffer artery. Increasing the ratio a/b represents a greater maximal distension. Decreasing the ratio a/b represents a smaller maximal distension.

#### Forcing function—the time domain blood pressure waveform

For proof of concept and validity testing one can use a Fourier series to represent blood pressure waveforms in these models [2]. A suitable and simple one for initial testing here is

(11) $$P_{a}=DBP+0.5PP+0.36PP\left[\sin\left(\omega t\right)+\frac{1}{2}\sin\left(2\omega t\right)+\frac{1}{4}\sin\left(3\omega t\right)\right]$$

for arterial pressure, P_a_, as a function of time, t, with ω being the angular frequency of the heartbeat, that is ω = 2πf for cardiac frequency, f, in Hz. Here SBP is systolic blood pressure, DBP is diastolic blood pressure, and PP is pulse pressure (SBP − DBP). In turn, the derivative of the arterial pressure waveform is

(12) $$\frac{dP_{a}}{dt}=0.36\omega \;\left[\cos\left(\omega t\right)+\cos\left(2\omega t\right)+\frac{3}{4}\cos\left(3\omega t\right)\right]\text{.}$$

Combining the cuff compliance, pressure-volume functions for the artery, and the arterial pressure waveform, one can write a set of equations for the rate of change in cuff pressure during an oscillometric pressure measurement in terms of P_0_, r, V_a_, C_cuff_, and time. We must work with the time derivative of cuff pressure, rather than absolute cuff pressure, because the compliance of the cuff and also the form of the artery volume vs. pressure function vary with time and pressure during a run. Cuff pressure can then be computed numerically by integrating equation (1a),

(13) $$\frac{dP}{dt}≅-r+\left(\frac{P_{0}+760-rt}{V_{0}}\right)\frac{dV_{a}\left(t\right)}{dt}\text{.}$$

Using the chain rule of calculus, and taking transmural pressure as arterial blood pressure minus cuff pressure,

(14) $$\frac{dV_{a}}{dt}=aV_{a0}e^{a\left(P_{a}-P_{0}+rt\right)}\cdot \left(\frac{dP_{a}}{dt}+r\right)\;{\text{for P}}_{\text{a}}–{\text{P}}_{0}+\text{rt}<0$$

(15) $$\frac{dV_{a}}{dt}=aV_{a0}e^{-b\left(P_{a}-P_{0}+rt\right)}\cdot \left(\frac{dP_{a}}{dt}+r\right)\;{\text{for P}}_{\text{a}}–{\text{P}}_{0}+\text{rt}\geq 0\text{,}$$

with artery pressure, P_a_, and its time derivative given by equations (5). Combining equations (1a) and (6) gives a precise model for cuff pressure oscillations.

#### Initial conditions

*Artery dimensions:* As a standard normal model consider a brachial artery with internal radius of 0.1 cm under zero distending pressure. The resting artery volume is V_a0_ = πr^2^L or

(16) $$V_{a0}=3.14\cdot {\left(0.1\;cm\right)}^{2}\cdot 10\;cm=0.3\;cm^{3}\text{.}$$

*Stiffness constant a:* For collapse to 10 percent at −20 mmHg transmural pressure we have

(17) $$a=\frac{\ln\left(0.1\right)}{P_{c}}=\frac{-2.3}{-20\;mmHg}=0.11\;mmHg^{-1}\text{.}$$

*Stiffness constant b:* It is easy to estimate the normal pressure compliance of the brachial artery in humans, C_n_ , from experiments using ultrasound. For example, using the data of Mai and Insana [14], the brachial artery strain (Δr/r) during a normal pulse is 4 percent for a blood pressure of 130/70 mmHg with pulse pressure 60 mmHg. In turn the volume of expansion during a pulse is 2πrΔrL, where r is the radius and L is the length of the compressed artery segment. Hence for a normal pressure radius of 0.2 cm the change in volume would be

(18) $$\Delta V_{a}=6.28\cdot 0.2cm\cdot 0.04\cdot 0.2cm\cdot 10cm=0.10\;cm^{3}\text{.}$$

The normal pressure compliance for the artery segment is the volume change divided by pulse pressure or

C_n_ = 0.10 ml / 60 mmHg = 0.0016 ml/mmHg.

For normal artery the pressure halfway between systolic and diastolic pressure, P_mid_ , would be 100 mmHg, so

(19) $$b=\frac{-\ln\left(\frac{C_{n}}{aV_{a0}}\right)}{P_{\mathrm{mid}}}=\frac{-\ln\left(\frac{0.0016\frac{cm^{3}}{mmHg}}{\frac{0.11}{mmHg}\cdot 0.3\;cm^{3}}\right)}{100\;mmHg}=0.03\;mmHg^{-1}\text{.}$$

Jeon et al. [13] working with a similar model used a = 0.09 mmHg^-1.^, b = 0.03 mmHg.

#### Numerical methods

In this model equations (1), (5), and (6) govern the evolution of cuff pressure as a function of time during cuff deflation. Equation (1) can be integrated numerically using techniques such as the simple Euler method coded in Microsoft Visual Basic, Matlab, or “C”. In the results that follow cuff deflation is started from a maximal level of 150 mmHg and continues over a period of 40 sec. Pressures are plotted every 1/20^th^ second. To extract the small oscillations from the larger cuff pressure signal, as would be done in an automatic instrument by an analog high pass filter, cuff pressure at time, t, is subtracted from the average of pressures recorded between times t − Δt/2 and t + Δt/2 , where Δt is the period of the pulse. For simplicity, filtered oscillations are not computed for time points that are Δt/2 seconds from the beginning or from the end of the time domain sample.

### Methods Part 2: Interpreting cuff pressure oscillations

Given this model and the associated insight into the physics of cuff pressure oscillations, one can also devise a scheme for estimating true systolic and diastolic blood pressures from an observed time domain record of cuff pressure and filtered cuff pressure oscillations. The method is based upon the ability, just described, to predict the amplitude of pulse pressure oscillations for a given diastolic pressure and pulse pressure and the ability to deduce exponential constants, a and b, from the rising and falling regions of the oscillation amplitude envelope. Details are as follows.

#### Artery motion during cuff deflation

**Figure 4:**
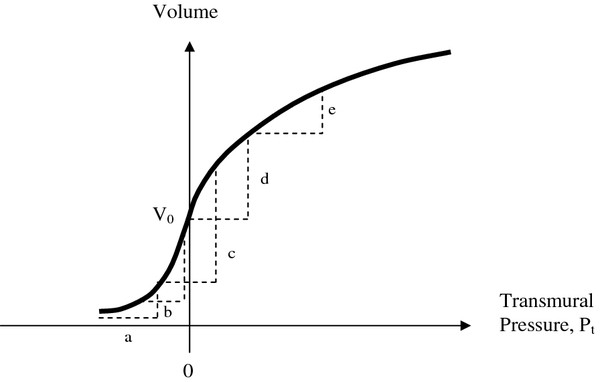
**Pressure-volume relationship for an artery (solid curve) including positive and negative transmural pressures.** Dashed triangles have equal bases indicating the range of transmural pressure (internal artery blood pressure minus cuff pressure) that determines the change in volume with each pulse. (**a**) Cuff pressure well above systolic with net distending pressure always negative. (**b**) Cuff pressure close to systolic. (**c**) Cuff pressure near mean arterial pressure with maximal volume changes. (**d**) Cuff pressure just below diastolic. (**e**) Cuff pressure well below diastolic.

The shape of the volume vs. pressure curve for arteries determines the driving signal for cuff pressure oscillations during an oscillometric measurement, as shown in Figure 4.

The pulsatile component of transmural pressure causes the artery to change in volume with each heartbeat. The magnitude of the change in transmural pressure is always equal to the pulse pressure (say, 40 mmHg) which is assumed to be constant during cuff deflation. As cuff pressure gradually decreases from well above systolic to well below diastolic pressure, the range of transmural pressure, P_t_, experienced by the artery changes. At (a) cuff pressure is well above systolic and net distending pressure is always negative. There is a small change in arterial volume because the artery becomes less collapsed as each arterial pulse makes the transmural pressure less negative. As cuff pressure approaches systolic the relative unloading of negative pressure becomes more profound. Because of the exponential shape of the arterial pressure-volume curve, the amount of volume change accelerates. At (b) cuff pressure is close to systolic. After this point the volume change continues to increase but at a decelerating rate, because of the shape of the pressure-volume curve. Hence (b) is the inflection point for systolic pressure. At (c) cuff pressure is near mean arterial pressure and the volume change is maximal. At (d) cuff pressure is just below diastolic. After this point, as shown in (e), the volume change becomes less and less with each pulse as the increasingly distended artery becomes stiffer. Hence (d) is the inflection point for diastolic pressure. Thus the nonlinear compliance of arteries and the shape of the arterial pressure-volume curve govern the amplitude of cuff pressure oscillations.

The particular volume change of the artery from the nadir of diastolic pressure to the subsequent peak of systolic pressure can be specified analytically from Equations (4a) and (4b) as follows. Consider P_t_ as the transmural pressure at the diastolic nadir of the arterial blood pressure wave and let PP be the pulse pressure. One can imagine three domains of transmural pressure. In Domain (1) P_t_ + PP < 0. In Domain (2) P_t_ < 0 and P_t_ + PP ≥ 0. In Domain (3) P_t_ > 0. The largest artery volume oscillations occur in Domain (2) when transmural pressure oscillates between positive and negative values. Doman (1) represents the head of the oscillation envelope in time, and Domain (3) represents the tail.

Using equations (4), the artery volume changes during the rising phase of the arterial pulse in each of the three domains are

Domain (1):

(20) $$\Delta V_{a}=V_{a0}\left[e^{a\left(P_{t}+PP\right)}-e^{aP_{t}}\right]$$

Domain (2):

(21) $$\Delta V_{a}=V_{a0}\left[1+\frac{a}{b}\left(1-e^{-b\left(P_{t}+PP\right)}\right)-e^{aP_{t}}\right]$$

Domain (3):

(22) $$\Delta V_{a}=V_{a0}\left[\frac{a}{b}\left(1-e^{-b\left(P_{t}+PP\right)}\right)-\frac{a}{b}\left(1-e^{-bP_{t}}\right)\right]\text{,}$$

where for cuff pressure, P, systolic blood pressure SBP, and diastolic blood pressure DBP, the transmural pressure P_t_ = DBP − P, and the pulse pressure PP = SBP − DBP.

It is easy to show by differentiating expressions (7) for Domains (1), (2), and (3) that the systolic and diastolic pressure points correspond exactly to the maximal and minimal slopes d(ΔV_a_)/dP_t_. Therefore a simple analysis for finding systolic and diastolic pressures points would involve taking local slopes of the oscillation envelope vs. pressure function. Slope taking, however, is vulnerable to noise in practical applications. An alternative approach that does not involve slope taking creates a model of each individual subject’s arm in terms of exponential constants a and b and then numerically finds the unique combination of systolic and diastolic arterial pressures that best reproduces the observed oscillation envelope.

#### Regression analysis for exponential constants

To obtain exponential constant, a, note that in the leading edge of the amplitude envelope at pressures near systolic blood pressure in Domain (1) the pulsatile change in cuff pressure is

(23) $$\begin{array}{ll} \Delta P & =\frac{\Delta V_{a}}{C_{\mathrm{cuff}}}=\frac{V_{a0}}{C_{\mathrm{cuff}}}\left[e^{a\left(P_{t}+PP\right)}-e^{aP_{t}}\right]\\ & =\frac{V_{a0}}{C_{\mathrm{cuff}}}\left[e^{a\left(PP\right)}-1\right]\;e^{aP_{t}}\\ & =\frac{V_{a0}}{C_{\mathrm{cuff}}}\left[e^{a\left(PP\right)}-1\right]\;e^{a\left(DBP-P\right)}\\ & =\frac{V_{a0}}{C_{\mathrm{cuff}}}\left[e^{a\left(PP\right)}-1\right]\;e^{a\left(DBP\right)}e^{-aP}=k_{1}e^{-aP} \end{array}$$

for constant, k_1_, during a cuff deflation scan in which cuff pressure, P, varies and the other variables are constant. (Note that here C_cuff_ is very nearly constant because the rising phase of the pulse happens in a very short time, roughly 0.1 sec.) Hence, $\ln\left(\Delta P\right)=\ln\left(k_{1}\right)-a\;P$, and a regression plot of the natural logarithm of the amplitude of pulse oscillations in the leading region of the envelope versus the instantaneous cuff pressure, P, yields a plot with slope − a. Thus we can obtain by linear regression an estimate of stiffness constant, a, as $\hat{a}=slope_{1}$. The range of the rising phase of the oscillation envelope from the beginning of the envelope to the first inflection point (maximal slope) can be used for the first semi-log regression. More simply, the range of the rising phase of the oscillation envelope from its beginning to one third of the peak height provides reasonable estimates of slope_1_.

Similarly in Domain (3) during the tail region of the amplitude envelope at cuff pressures less than the maximal negative slope of the falling phase

(24) $$\begin{array}{ll} \Delta P & =\frac{\Delta V_{a}}{C_{\mathrm{cuff}}}=\frac{V_{a0}}{C_{\mathrm{cuff}}}\left[\frac{a}{b}\left(1-e^{-b\left(P_{t}+PP\right)}\right)-\frac{a}{b}\left(1-e^{-bP_{t}}\right)\right]\\ & =\frac{V_{a0}}{C_{\mathrm{cuff}}}\frac{a}{b}\left[1+e^{-b\left(PP\right)}\right]\;e^{-bP_{t}}\\ & =\frac{V_{a0}}{C_{\mathrm{cuff}}}\frac{a}{b}\left[1+e^{-b\left(PP\right)}\right]\;e^{-b\left(DBP-P\right)}=k_{3}e^{\mathrm{bP}} \end{array}$$

hence, $\ln\left(\Delta P\right)=\ln\left(k_{3}\right)+b\;P$, and a regression plot of the natural logarithm of the amplitude of pulse oscillations in the envelope tail versus cuff pressure at the time of each pulse yields a plot with slope b. In turn, we can obtain by linear regression an estimate of stiffness constant, b, as $\hat{b}=slope_{3}$. The range of the falling phase of the oscillation envelope from the second inflection point (maximal negative slope) of the oscillation envelope to the end of the envelope can be used to define the range of the second semi-log regression. More simply, the range of the falling phase of the oscillation envelope from two thirds of the peak height to the end of the envelope provides reasonable estimates of slope_3_. The slope estimates from the head and tail regions of the amplitude envelope include multiple points and so are relatively noise resistant. Other variables involved in the lumped constants, k_1_ and k_3_, are not relevant to the estimation of exponential constants a and b.

#### Least squares analysis

Having estimated elastic constants a and b for a particular envelope of oscillations from a particular patient at a particular time, it is straightforward in a computer program to find SBP and DBP values that reproduce the observed envelope function most faithfully. Let y(P) be the observed envelope amplitude as a function of cuff pressure, P, and let y_max_(P_max_) be the observed peak amplitude of oscillations at cuff pressure P_max_. Let $\hat{y}$(P, SBP, DBP) be the simulated envelope amplitude as a function of cuff pressure, P, for a particular pulse and a particular test set of systolic and diastolic pressure levels. The values of $\hat{y}$ are obtained from equations (7) and the prevailing cuff compliance as follows

Domain (1):

(25) $$\hat{y}=\frac{\Delta V_{a}}{C_{\mathrm{cuff}}}=V_{a0}\left[e^{a\left(SBP-P\right)}-e^{a\left(DBP-P\right)}\right]\cdot \frac{P+760}{V_{0}}$$

Domain (2):

(26) $$\hat{y}=\frac{\Delta V_{a}}{C_{\mathrm{cuff}}}=V_{a0}\left[1+\frac{a}{b}\left(1-e^{-b\left(SBP-P\right)}\right)-e^{a{\left(DBP-P\right)}_{t}}\right]\cdot \frac{P+760}{V_{0}}$$

Domain (3):

(27) $$\hat{y}=\frac{\Delta V_{a}}{C_{\mathrm{cuff}}}=V_{a0}\left[\frac{a}{b}\left(1-e^{-b\left(SBP-P\right)}\right)-\frac{a}{b}\left(1-e^{-b\left(DBP-P\right)}\right)\right]\cdot \frac{P+760}{V_{0}}\text{.}$$

Let $\hat{y}$_max_(P_max_, SBP, DBP) be the predicted peak of the oscillation envelope at cuff pressure P_max_ . A figure of merit for goodness of fit between modeled and observed oscillations for particular test values of SBP and DBP is the sum of squares over all measured pulses

(28) $$SS\left(SBP,DBP\right)=\sum_{all\;pulses}{\left(\frac{y}{y_{\max}}-\frac{\hat{y}}{{\hat{y}}_{\max}}\right)}^{2}\text{.}$$

The values of SBP and DBP that minimize this sum of squares are the taken as the best estimates of systolic and diastolic pressure by the oscillometric method.

Here cuff pressure, P, is the cuff pressure at the time of each oscillation. Use of the amplitude normalized ratios y/y_max_ and $\hat{y}$/$\hat{y}$_max_, means that it is not necessary to know the zero pressure volume of the artery, V_a0_ , or cuff volume V_0_, which depend on anatomy and geometry of a particular arm and cuff and are constants. It is the shape of the amplitude envelope in the pressure domain that contains the relevant information. The least squares function, SS, includes information from all of the measured oscillations and so is relatively noise resistant.

A variety of numerical methods may be used to find the unique values of SBP and DBP corresponding to the minimum sum of squares. Here, to demonstrate proof of concept, we evaluate the sum of squares, SS, over a two-dimensional matrix of candidate systolic and diastolic pressures at 1 mmHg intervals and identify the minimum sum of squares by plotting. The values of SBP and DBP corresponding to this minimum sum of squares are the best fit estimates for a particular oscillometric pressure run. The best fit model takes into account the prevailing artery stiffness and also the prevailing pulse pressure.

## Results and discussion

### Normal model

Particular parameter values for the standard normal model are as shown in Table 1.

**Table 1:** Standard parameters for the oscillometric blood pressure model

**Parameter**	**Definition**	**Value**	**Units**
P_0_	Cuff pressure at onset of deflation	150	mmHg
r	Cuff pressure decay rate	3	mmHg/sec
PP	Arterial pulse pressure	40	mmHg
f	Cardiac frequency (heart rate)	80	beats/min
V_a0_	Artery segment volume at zero pressure	0.3	ml
a	Exponential constant	0.03	1/mmHg
b	Exponential constant	0.11	1/mmHg
C_n_	Artery segment compliance at 100 mmHg pressure	0.0016	ml/mmHg

**Figure 5:**
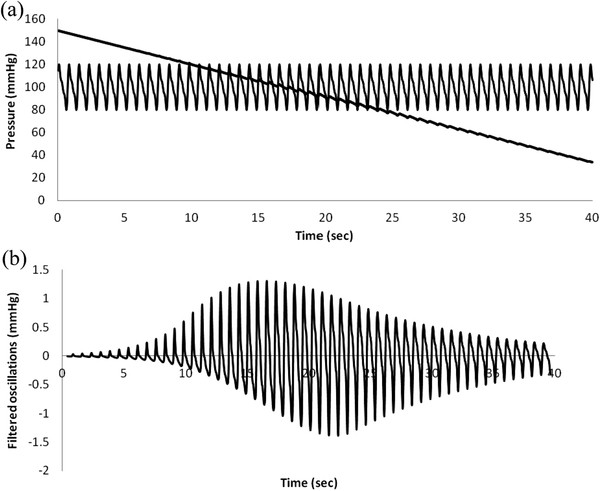
**Simulated oscillometric blood pressure determination in a normal patient.** (**a**) Blood pressure and cuff pressure vs. time. (**b**) High pass filtered cuff pressure oscillations.

**Figure 6:**
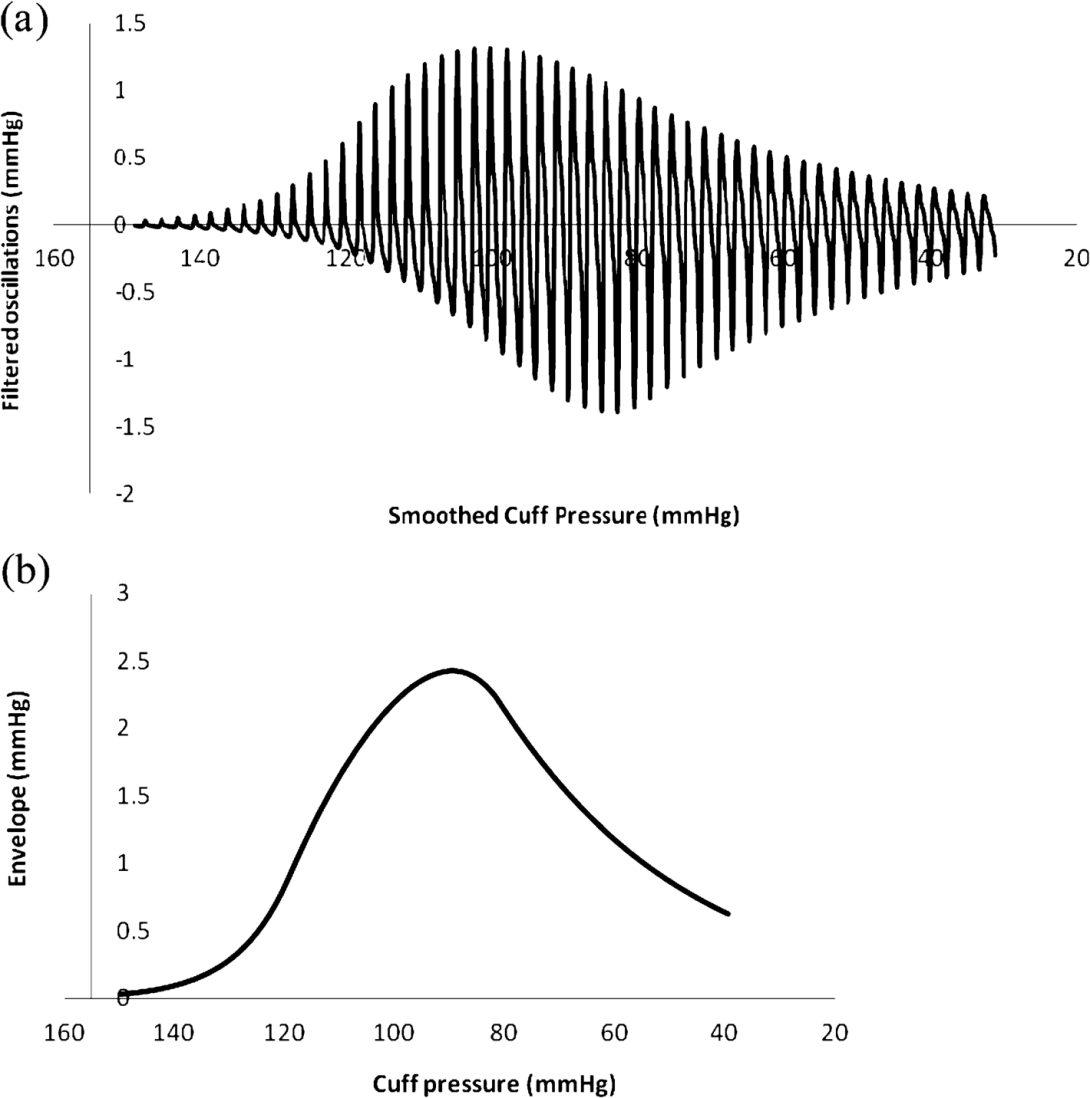
**Simulated oscillometric blood pressure determination in a normal patient.** (**a**) Cuff pressure oscillations vs. pressure. (**b**) Amplitude envelope obtained from maximum minus minimum cuff pressure over each heartbeat.

Figures 5 (a) and (b) show plots of cuff pressure and arterial pressure vs. time and high pass filtered cuff pressure oscillations vs. time. Figure 6 shows cuff pressure oscillations vs. cuff pressure and the amplitude envelope of cuff pressure for the standard normal model. Cuff pressure oscillations were obtained by subtracting each particular value from the moving average value over a period of one heartbeat.

### Varying arterial compliance

**Figure 7:**
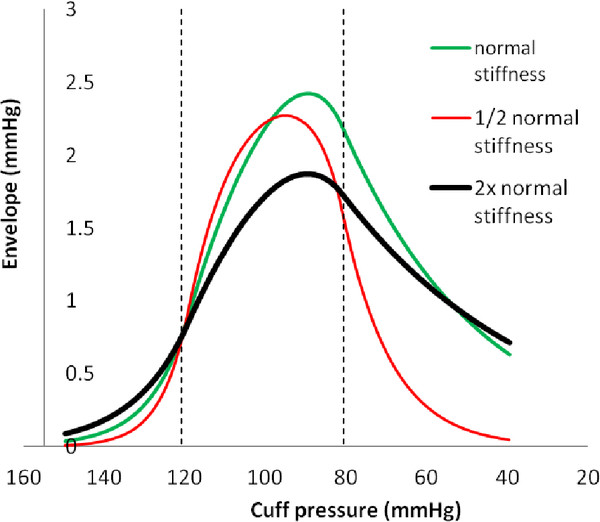
**Amplitude envelopes for varying arterial stiffness.** Stiffness is represented as inverse compliance. Exponential constants a and b for 1/2 normal stiffness are multiplied by ln(2) = 1.44. Exponential constants a and b for 2x normal stiffness are divided by 1.44. In all cases actual blood pressure was 120/80 mmHg.

Prior studies have suggested that variations in arterial wall stiffness and arterial pulse pressure cause errors in systolic and diastolic blood pressure estimates using the oscillometric method [4,14,15]. Hence, these variables were studied explicitly. Figure 7 shows effects of varying arterial stiffness, represented by the constants a and b in the bi-exponential artery model. Actual blood pressure was 120/80 mmHg. The cuff oscillation ratios for systolic pressure are similar with varying stiffness. However, the cuff oscillation ratios for diastolic pressure differ greatly among more compliant, normal, and stiffer arteries, indicating that the same oscillation ratios cannot be used to determine diastolic pressures from the amplitude envelope when artery stiffness varies. The diastolic oscillation ratios decrease from about 94% to 88% to 75% as stiffness decreases from high to normal to low. Oscillation amplitude ratios for diastolic pressure in particular are highly dependent upon the stiffness of arteries. Since artery stiffness varies with age, this phenomenon may be a problem clinically. Note, however, that the maximum and minimum slopes of the envelope in the pressure domain still correlate well with true systolic and diastolic pressures.

### Varying pulse pressure

**Figure 8:**
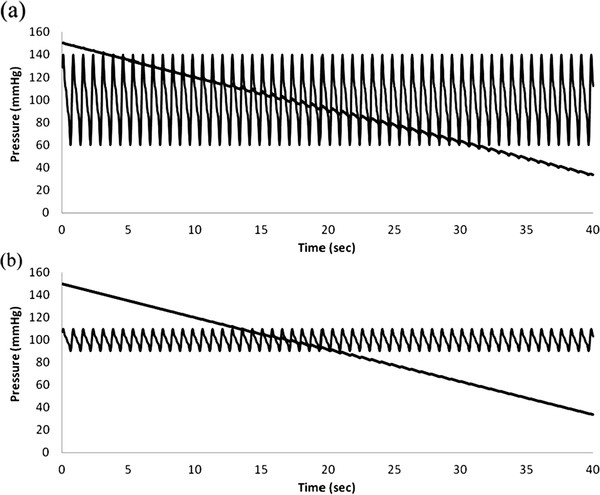
**Simulations of varying arterial pulse pressure.** (**a**) and (**b**) blood pressure and cuff pressure vs. time, 140/60 mmHg vs. 110/90 mmHg.

**Figure 9:**
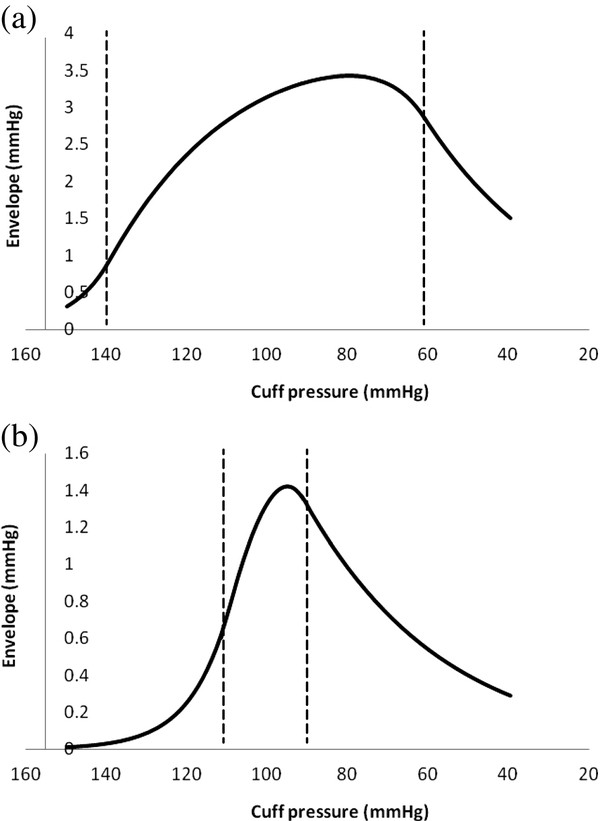
**Simulations of varying arterial pulse pressure.** (**a**) and (**b**) amplitude envelopes for 140/60 mmHg vs. 110/90 mmHg.

Figures 8 and 9 show raw data and amplitude oscillation envelopes for cases of high and low pulse pressure. The amplitude of cuff pressure oscillations is greater for widened pulse pressure than for narrowed pulse pressure. The shape of the amplitude envelope is distorted for widened pulse pressure; however the maximum and minimum slopes of the envelope in the pressure domain still correlate well with true systolic and diastolic pressures. Characteristic ratios for systolic and diastolic pressures vary with pulse pressure. The characteristic ratio for systolic pressure is substantially smaller for widened pulse pressure and significantly larger for narrowed pulse pressure. The characteristic ratio for diastolic pressure is substantially larger for widened pulse pressure than for narrowed pulse pressure.

### Regression analysis for systolic and diastolic pressures

**Figure 10:**
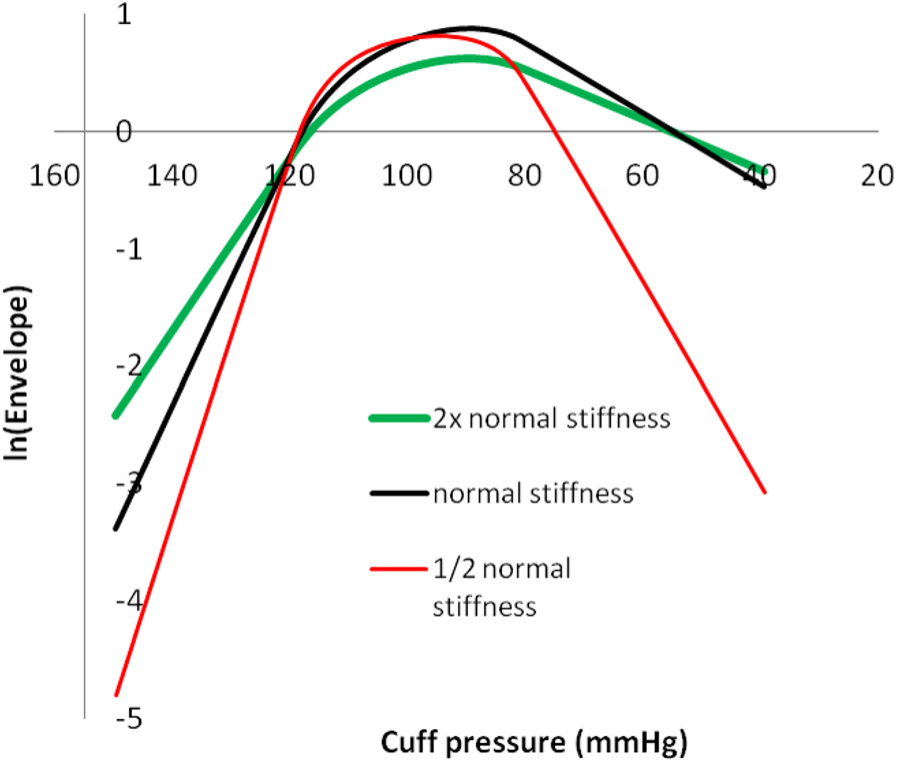
**Semi-log plots for determining model constants from amplitude envelope data.** Note straight line regions in rising and falling phases of the curves.

Figure 10 shows semi-log plots for the envelope functions shown in Figure 7 representing arteries of varying stiffness. The linear portions of the plots in the head and tail regions of log envelope amplitude vs. cuff pressure curves are evident. The artery stiffness constants a and b obtained from linear regression slopes for these head and tail regions are close to the nominal input values (data in Table 2).

**Table 2:** Validation of algorithm for estimation of systolic and diastolic pressures

**Scenario and parameter**	**Actual value**	**Algorithm value**
***Normal***		
Constant a (mmHg^-1^)	0.11	0.105
Constant b (mmHg^-1^)	0.03	0.0311
SBP (mmHg)	120	119
DBP (mmHg)	80	80
***Twice normal stiffness***		
Constant a (mmHg^-1^)	0.076	0.074
Constant b (mmHg^-1^)	0.021	0.0214
SBP (mmHg)	120	119
DBP (mmHg)	80	80
***Half normal stiffness***		
Constant a (mmHg^-1^)	0.158	0.155
Constant b (mmHg^-1^)	0.0432	0.043
SBP (mmHg)	120	119
DBP (mmHg)	80	80
***Half normal pulse pressure***		
Constant a (mmHg^-1^)	0.110	0.108
Constant b (mmHg^-1^)	0.030	0.030
SBP (mmHg)	110	110
DBP (mmHg)	90	89
***Twice normal pulse pressure***		
Constant a (mmHg^-1^)	0.11	0.105
Constant b (mmHg^-1^)	0.03	0.030
SBP (mmHg)	140	138
DBP (mmHg)	60	60

**Figure 11:**
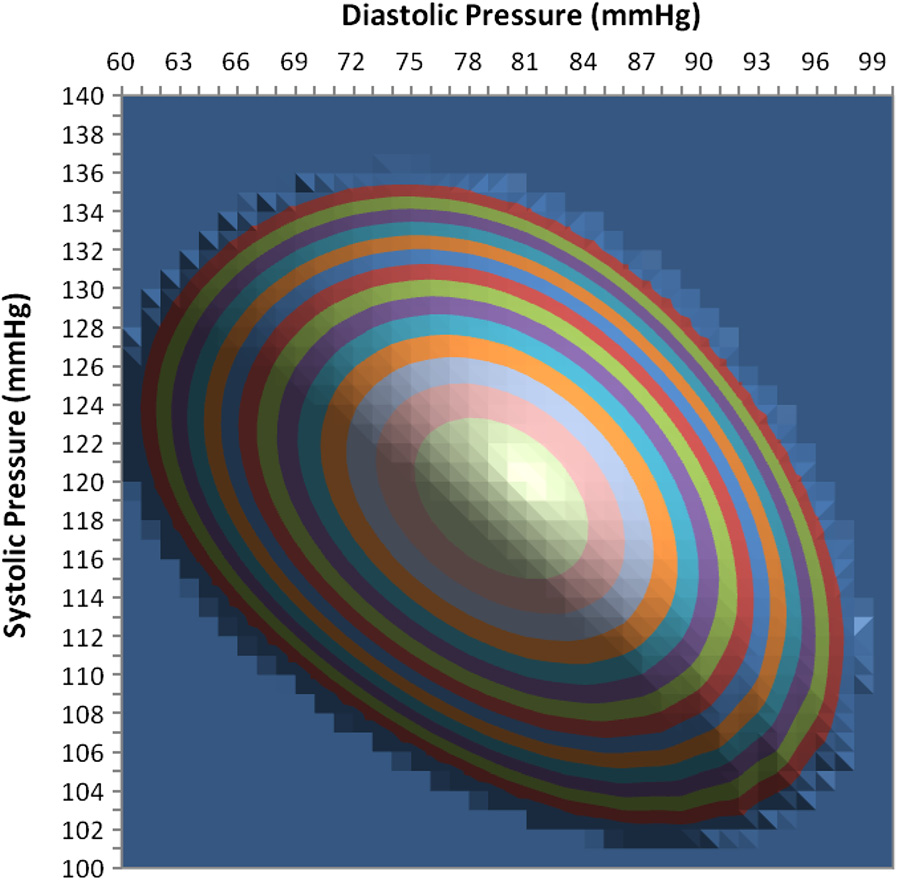
**Contour plot of sum of squares goodness of fit measure showing a minimum value and best agreement at an estimated blood pressure of 119/80 mmHg, evaluated for input data computed with known pressure of 120/80 mmHg.** Flat background indicates exceedingly large, off-scale sums of squares.

Figure 11 shows a contour map of the sum of squares function in equation (11) for different test values of systolic and diastolic blood pressure using the previously determined regression values for stiffness constants a and b. The semi-log regression slopes give values for constants a and b of 0.1074 and 0.0303, respectively, versus the actual values of 0.110 and 0.030 used in the model to create the analyzed cuff pressure oscillations. The minimum sum of squares indicates the best fit between the oscillation envelope predicted by the mathematical model and the observed oscillation envelope. The minimum sum of squares is shown in Figure 11 as the center of the target-like pattern of colored, equal value contours. This point indicates the least squares solutions both for systolic pressure on the vertical scale and for diastolic pressure on the horizontal scale. Larger diameter ring-shaped contours indicate progressively greater sums of squares and therefore progressively greater disagreement between observed and predicted oscillation envelopes. The contour interval is 0.1 dimensionless units. The flat background indicates exceedingly large, off-scale sums of squares > 1.5 units. The minimum sum of squares occurs for test values SBP/DBP of 119/80 mmHg. The actual pressure was 120/80 mmHg.

**Figure 12:**
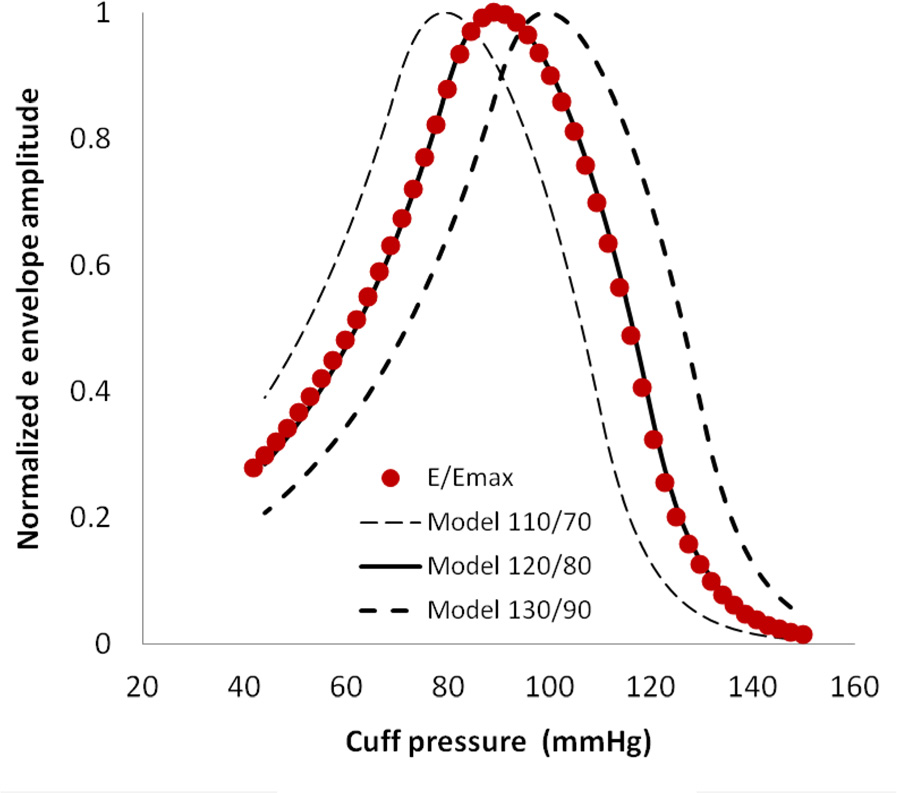
Agreement of model (curves) and input (filled circles) amplitude functions in the normal pressure case.

Figure 12 illustrates the sensitivity of the reconstruction algorithm to differences between various test levels and the actual values of systolic and diastolic blood pressure, in this case 120/80 mmHg. A low value of test pressure (110/70) creates a reconstructed envelope (dashed curve to left) that is clearly discordant with the observed normalized envelope values, E/E_max_, shown as filled circles. A high value of test pressure (130/90) leads to equally discordant reconstructions in the opposite direction (heavy dashed curve to right). For both low and high test values the sum of squared differences is obviously large. The reconstructed model for the actual pressure (120/80) is shown as the solid curve. This illustration demonstrates the sensitivity of the least squares approach.

### Validation of the regression procedure

The cuff-arm-artery model of an oscillometric pressure measurement described in Methods Part One can be used to validate the regression and analysis procedure of Methods Part Two. An unlimited number and wide variety of test scenarios can be simulated in the model as unknowns for testing by the regression scheme, including a wide range of arterial stiffnesses and a wide range of pulse pressures, heart rates, blood pressure waveforms, cuff sizes, arm sizes, cuff lengths, artery diameters, etc. Importantly, the regression analysis assumes no prior knowledge of these model parameters or of the blood pressure used to generate the simulated oscillations. Cuff pressure oscillations and absolute cuff pressure are the only inputs to the algorithm for obtaining systolic and diastolic pressures.

The data summarized in Table 2 show the effectiveness of the proposed regression procedure in small sample of various possible test scenarios, including varying artery stiffness and varying pulse pressure. This small, systematic sample includes challenging cases for the algorithm. The accuracy is quite satisfactory, with reconstructed pressures within 0, 1, or 2 mmHg of the actual pressures in the face of varying artery stiffness and varying pulse pressure. The root mean squared error is $\sqrt{8}/10$ = 0.28 mmHg.

## Discussion

The challenge of creating a satisfactory theoretical treatment of the genesis and interpretation of cuff pressure oscillations has attracted a diverse community of thinkers [4,5,7-10,16]. Nevertheless, specifying a valid method for extracting systolic and diastolic pressures from the envelope of cuff pressure oscillations remains an open problem. Here is presented a mathematical model incorporating anatomy, physiology, and biomechanics of arteries that predicts cuff pressure oscillations produced during noninvasive measurements of blood pressure using the oscillometric method. Understanding of the underlying mechanisms leads to a model-based algorithm for deducing systolic and diastolic pressures accurately from cuff pressure oscillations in the presence of varying arterial stiffness or varying pulse pressure.

The shape of the oscillation amplitude envelope dictates the stiffness parameters for the artery during both compression and distension. Semilog regression procedures give good estimates of the artery stiffness parameters that characterize each individual cuff deflation sequence. Using these parameters one can create and exercise an individualized cuff-arm-artery model for a wide range of possible systolic and diastolic pressures. The pair of systolic and diastolic pressures that best reproduces the observed oscillation envelope according to a least squares criterion constitutes the output of the algorithm.

When applied to amplitude normalized oscillation data the algorithm is insensitive to variations among subjects in zero pressure artery volume, V_a0_ , or initial cuff volume V_0_, since these terms are constants that are eliminated by the normalization procedure. Compression of the entire length of artery underlying the cuff is not necessary. Incomplete coupling of cuff pressure to the artery near the ends of the cuff merely decreases the ratio V_a0_ / V_0_ without effecting the extracted systolic and diastolic pressures.

The cuff-arm-artery model can be used as well to test the validity of the algorithm for over a wide range of possible conditions by generating trial cuff pressure data for known arterial pressure waveforms. A stress test for the algorithm can be done by comparing systolic and diastolic pressure levels extracted from synthesized cuff pressure oscillations with the arterial pressure that generated the synthesized oscillations over a wide range of test conditions. These conditions may include extreme cases that are hard to reproduce experimentally, contamination with excessive noise, any conceivable blood pressure waveforms, cardiac arrhythmias such as atrial fibrillation, etc. Such computational experiments, in addition to future animal and clinical studies, can boost confidence in the reliability of the oscillometric method and can suggest further refinements.

Here for convenience we have used the bi-exponential model to generate cuff pressure oscillations for algorithm testing. However, the regression algorithm does not “know” where the sample data came from. It tries to extract constants a and b from the head and tail portions of the semi-log plot of oscillation amplitude versus cuff pressure. The resulting best fit values of a and b will still work for non-ideal or noise contaminated data to produce a model envelope that can be matched to the actual data. An extremely stiff artery with a linear pressure volume curve is easily accommodated by this process, since e^x^ ≈ 1 + x for small values of x. In this limiting case the exponential pressure-volume curve becomes linear. An exceptionally flabby artery, rather like dialysis tubing, is well described by larger values of a and b and a larger ratio a/b. Thus the family of bi-exponential models is very inclusive of a wide range of arterial mechanical properties, as suggested in Figure 3.

Classically the oscillometric method has been relatively well validated as a measure of mean arterial pressure, which is indicated by the peak of the oscillation amplitude envelope [4]. Automated oscillometric pressure monitors have found use in hospitals for critical care monitoring in which the goal is to detect any worrisome trend in blood pressure more so than the exact absolute value. Out of hospital use of the oscillometric method in screening for high blood pressure is more problematic, because heretofore the accuracy of systolic and diastolic end points has been questioned and doubted. For example Stork and Jilek [17] studied two published algorithms, differing in detail and based on cuff oscillation ratios of either 50% for systolic and 80% for diastolic or 40% for systolic and 55% for diastolic. Compared to a reference pressure of 122/78 mmHg the algorithmic methods applied to oscillometric data gave pressures of 135/88 and 144/81 mmHg, respectively. An advisory statement from the Council for High Blood Pressure Research, American Heart Association [18] stressed the need for caution in the selection of all instruments used for blood pressure determination and the need for continuing studies to validate their the safety and reliability.

Accurate measurements of blood pressure in routine clinic and office settings are important because systemic arterial hypertension is a major cause of serious complications, including accelerated atherosclerosis, heart attacks, strokes, kidney disease, and death. These serious complications increase smoothly with every point above the nominal 120/80 mmHg, hence even small increases in blood pressure are important to detect. In screening for hypertension systematic bias or inaccuracy in blood pressure readings of a few mmHg can be significant, since the difference between high normal (85 diastolic) and abnormal (90 diastolic) is only a few mmHg. A recent 1 million-patient meta-analysis suggests that a 3–4 mmHg increase in systolic blood pressure would translate into 20% higher stroke mortality and a 12% higher mortality from ischemic heart disease [19].

False negative readings would be problematic because untreated high blood pressure can lead to strokes, blindness, kidney failure, and lethal heart attacks. False positive readings would be undesirable because the usual drugs for hypertension must be taken every day for life and can be expensive. They also have side effects. Hence accurate readings are essential. Given a reliable algorithm for extracting systolic and diastolic pressures, an automatic oscillometric device could provide screening for high blood pressure that is performed in the same way each time without inter-observer variation. The present research could lead to a wider role for oscillometric blood pressure monitors in physicians’ offices and clinics.

## Conclusions

The analytical approach and algorithm presented here represent a solution to an open problem in biomedical engineering: how to determine systolic and diastolic blood pressures using the oscillometric method. Current algorithms for oscillometric blood pressure implemented in commercial devices may be quite valid but are closely held trade secrets and cannot be independently validated. The present paper provides a physically and physiologically reasonable approach in the public domain that can be independently criticized, tested, and refined. Future demonstration of real-world accuracy will require data comparing oscillometric and intra-arterial pressures in human beings over a range of test conditions including variable cuff size, arm diameter, cuff tightness, cuff deflation rate, etc. Further development and incorporation of this algorithm into commercial devices may lead to greater confidence in the accuracy of systolic and diastolic pressure readings obtained by the oscillometric method and, in turn, an expanded role for these devices.

## Competing interests

The author declares that he has no competing interests.

## Author’s contributions

CB is the only author and is responsible for all aspects of the research and the intellectual and technical content of the manuscript.
